# DBS Direct Elution and Multiplex Digital PCR for *SMN1* and *SMN2* Copy-Number and *CFTR* Variant Detection: Development and Validation of a Molecular Screening Workflow

**DOI:** 10.3390/diagnostics16213538

**Published:** 2026-10-28

**Authors:** Eleftheria Dikoglou-Tzanetatou, Iro Skopa, Myrto Poulou, Elisavet Kouvidi, Maria Grigoriadou, Eleni Leiletzoglou, Aggeliki Kolialexi

**Affiliations:** Department of Genetics, Institute of Child Health, 11527 Athens, Greece; e.ditza@ich.gr (E.D.-T.); h.skopa@ich.gr (I.S.); m.poulou@ich.gr (M.P.); e.kouvidi@ich.gr (E.K.); mgrigoriadou@ich.gr (M.G.); e.leiletzoglou@ich.gr (E.L.)

**Keywords:** carrier screening, *SMN1*, *SMN2*, SMA, digital PCR (dPCR), *CFTR* p.Phe508del variant

## Abstract

**Background/Objectives**: Scalable molecular workflows may facilitate carrier screening for spinal muscular atrophy (SMA) and cystic fibrosis (CF). This study aimed to develop and validate a dried blood spot (DBS)-based multiplex digital PCR (dPCR) workflow for *SMN1* and *SMN2* copy-number analysis and targeted detection of *CFTR* p.Phe508del. **Methods**: The workflow combined direct DNA elution with multiplex dPCR. Analytical validation used 50 previously characterized samples, with multiplex ligation-dependent probe amplification (MLPA) and Sanger sequencing as reference methods. The workflow was subsequently applied to 1670 women from Crete and Ikaria, Greece, followed by targeted testing of 10 partners. **Results**: All 50 directly eluted samples amplified successfully. dPCR showed complete agreement with reference methods. Repeatability coefficients of variation were below 5%. Copy-number classification performance estimates are apparent, as thresholds were derived and evaluated in the same small validation cohort. Among 1670 women screened, 34 (2.04%) were identified as *SMN1* carriers and 20 (1.20%) as *CFTR* p.Phe508del carriers. Partner testing identified two carrier couples. **Conclusions**: The DBS-based multiplex dPCR workflow demonstrated complete analytical concordance with reference methods in this preliminary validation and was implemented in a population screening cohort. Its targeted scope leaves residual carrier risk, requiring appropriate counseling. Larger independent validation and formal throughput and cost assessments are needed before routine implementation.

## 1. Introduction

Carrier screening enables the identification of individuals at increased reproductive risk for severe inherited disorders, facilitating informed reproductive decisions and genetic counseling. Advances in molecular diagnostics have improved the feasibility of population-based screening, although implementation requires analytically reliable, scalable, and cost-effective workflows. Spinal muscular atrophy (SMA) and cystic fibrosis (CF) are among the more frequent severe autosomal recessive disorders for which carrier screening is recommended [1,2,3], with estimated carrier frequencies of approximately 1 in 25–30 for CF in populations of European ancestry [4] and 1 in 38–50 for SMA [2,5].

In Greece, SMA has been described as the third most common severe autosomal recessive disorder, after hemoglobinopathies and CF, with a reported carrier frequency of 1 in 43 and a minimum incidence of approximately 1 in 12,000 live births [5]. Hemoglobinopathies have been addressed through established prevention programs since the 1970s [6]. A Greek exome-sequencing study of 1000 predominantly clinically referred individuals identified high carrier frequencies for HBB (6.1%), HBA1/2 (5.5%), GJB2 (5.0%), and *CFTR* (4.2%), although the cohort was not population-based and *SMN1* copy-number alterations require complementary testing approaches [7]. Carrier screening for hemoglobinopathies and *CFTR* p.Phe508del is already reimbursed in Greece, whereas SMA carrier screening is not [7].

SMA is primarily caused by biallelic loss or pathogenic alteration in *SMN1*, while *SMN2* copy-number is an important modifier of disease severity [2]. In CF, p.Phe508del is the most common pathogenic *CFTR* variant, accounting for 53.4% of CF alleles in a study of 437 Greek patients [8], consistent with the 52.9% reported for Greece in an international analysis [9]. However, the original Greek study reported allele frequencies without specifying the proportion of patients carrying at least one p.Phe508del allele or being homozygous for this variant. The Greek *CFTR* mutation spectrum is heterogeneous, and a panel of 23 common variants has been estimated to detect fewer than 80% of carriers in Greece and Italy [4]. Reflecting the need for broader variant coverage, the 2023 American College of Medical Genetics and Genomics (ACMG) recommendations established a minimum panel of 100 *CFTR* variants for carrier screening [10].

National reproductive carrier screening programs have been established in countries such as Israel, where publicly funded screening has included CF since 2008 and was expanded in 2024 [11], and Australia, where Medicare-funded screening for CF, SMA, and fragile X syndrome has been available since 2023 [2,12]. More recently, Canadian recommendations have supported provincial screening programs targeting common severe inherited disorders [1]. No comparable nationwide reproductive carrier screening program for SMA and CF has been established in Greece. Implementation remains heterogeneous internationally, with challenges extending beyond analytical performance to program costs, genetic counseling, partner testing, and equitable access [4,13]. Dried blood spots (DBS) offer practical advantages for population screening through minimally invasive collection, simplified transport and storage, and centralized sample processing [14].

Digital PCR (dPCR) enables sensitive molecular detection and absolute quantification, making it particularly suitable for copy-number analysis [15]. DBS-based assays for *SMN1* copy-number determination have previously been described [16,17]. Combining direct DNA elution from DBS with multiplex dPCR may simplify sample preparation and enable simultaneous analysis of different genetic targets. However, the analytical performance of this approach requires further evaluation.

In this study, we developed and validated a DBS-based direct-elution multiplex dPCR assay for *SMN1* and *SMN2* copy-number analysis and targeted *CFTR* p.Phe508del detection. Analytical performance was assessed against multiplex ligation-dependent probe amplification (MLPA) and Sanger sequencing. The assay was subsequently applied to 1670 women from Crete and Ikaria, Greece, followed by targeted partner testing, to evaluate its implementation in a reproductive carrier screening pathway and determine the observed carrier frequencies.

## 2. Materials and Methods

### 2.1. Validation Samples and DBS Collection

Capillary blood samples were collected by finger prick using sterile, single-use safety lancets with a 1.8 mm penetration depth (Sarstedt AG & Co. KG, Nümbrecht, Germany). Blood was applied to Guthrie cards (Revvity, Waltham, MA, USA), ensuring saturation of all five preprinted circles. Cards were air-dried at room temperature for 3–4 h, transported to the laboratory in paper envelopes within 48 h, and individually wrapped in aluminum foil and stored at 4 °C until analysis.

Analytical validation included 50 previously characterized DBS samples collected between 2025 and 2026 from patients and their family members who had provided written informed consent for research use. Samples were purposively selected from archived specimens to represent different *SMN1* and *SMN2* copy-number states and include *CFTR* p.Phe508del-positive samples; they were neither randomly nor consecutively selected.

For *SMN1*, MLPA identified 3, 14, 27, and 5 samples with 0, 1, 2, and 3 copies, respectively, while one additional sample (C377) yielded an indeterminate result. For *SMN2*, the corresponding distribution was 5, 19, 23, and 3 samples. Eight samples were positive and 42 were negative for *CFTR* p.Phe508del. All validation samples were independent of the population screening cohort. Reference results were generated during this study using MLPA and Sanger sequencing on DNA extracted from the same DBS cards, as described in Section 2.5.

### 2.2. Dried Blood Spot Punching

Three DBS discs (3.2 mm in diameter) were obtained from each Guthrie card using the Panthera-Puncher™ 9 (Revvity, Waltham, MA, USA) and automatically transferred into 96-well plates for direct DNA elution and dPCR analysis. For reference-method analysis of the validation samples, seven to eight additional discs were collected from the corresponding cards and transferred into individual tubes for conventional DNA extraction.

### 2.3. DNA Elution and Extraction

For dPCR analysis, three DBS punches per sample were washed three times with 500 µL of TE buffer (10 mM Tris-HCl, 1 mM EDTA, pH 8.0). DNA was directly eluted by adding 50 µL of elution solution containing 47 µL of FTA buffer and 3 µL of proteinase K (Qiagen, Hilden, Germany). Samples were incubated at 60 °C for 25 min, followed by heating at 95 °C for 5 min. A 2 µL aliquot of the resulting eluate was used for dPCR without further purification.

For reference-method analysis, genomic DNA was extracted from additional punches obtained from the same validation DBS cards, although not necessarily from the same blood-spot circles, using the Maxwell^®^ RSC FFPE Plus DNA Kit (Promega, Madison, WI, USA) according to the manufacturer′s instructions.

DNA concentration and spectrophotometric purity (A260/A280 and A260/A230 ratios) were assessed in both preparations using a NanoDrop spectrophotometer (Thermo Fisher Scientific, Waltham, MA, USA) (Supplementary Tables S1 and S2). Measurements of crude eluates were interpreted descriptively, as DNA concentrations were not independently verified using a DNA-specific method. Amplifiable template was additionally assessed through *RPPH1* copy numbers derived from dPCR (Section 3.2).

### 2.4. Multiplex dPCR Assay and Quality Control

A multiplex dPCR assay was developed for *SMN1* and *SMN2* copy-number determination and targeted detection of *CFTR* p.Phe508del, using *RPPH1* as the reference target for copy-number determination and assay quality control. All primers and hydrolysis probes were custom-synthesized by Eurofins Genomics (Ebersberg, Germany), with their sequences and fluorophores provided in Supplementary Table S3.

A common primer pair was combined with *SMN1*- and *SMN2*-specific hydrolysis probes labeled with different fluorophores. Separate primer–probe mixes for *SMN1/SMN2*, *CFTR* p.Phe508del, and *RPPH1* were combined in a single 40 µL multiplex reaction. The compositions of the 10× assay mixes and final reaction mixture, containing QIAcuity Probe Master Mix (4×), EcoRI (New England Biolabs, Ipswich, MA, USA), nuclease-free water, and 2 µL of DNA template, are provided in Supplementary Tables S4 and S5, respectively.

Digital PCR was performed using the QIAcuity platform (Qiagen, Hilden, Germany) with 24-well nanoplates. Amplification consisted of initial denaturation at 95 °C for 2 min, followed by 40 cycles at 95 °C for 15 s and 60 °C for 30 s. Channel-specific imaging settings are provided in Supplementary Table S6.

Positive and negative partitions were identified using QIAcuity software (version 3.2.0.0; Qiagen, Hilden, Germany), which calculated absolute target concentrations. Fluorescence thresholds were initially assigned automatically and adjusted manually when necessary following visual inspection of amplitude plots. *SMN1* and *SMN2* copy numbers were determined relative to *RPPH1* using the classification thresholds described in Section 3.4, while *CFTR* p.Phe508del status was determined from the corresponding fluorescence channel.

Positive controls with known *SMN1/SMN2* copy numbers and *CFTR* p.Phe508del genotypes were included during assay development but not in every run. A no-template control (NTC) was included in each run, which was accepted only when the NTC showed no more than two positive partitions per fluorescence channel. Individual reactions were required to contain at least 18,000 valid partitions. Samples with fewer than 18,000 valid partitions or failure of *RPPH1* amplification were re-eluted and reanalyzed by dPCR.

During assay optimization, each target-specific primer–probe assay was initially evaluated in singleplex reactions, followed by testing in different multiplex combinations and finally in the complete four-target reaction (*SMN1*, *SMN2*, *CFTR* p.Phe508del, and *RPPH1*). No multiplex-specific interference was observed under the tested conditions.

### 2.5. MLPA and Sanger Sequencing Analysis

*SMN1* and *SMN2* copy-number analysis was performed using the SALSA MLPA Probemix P021-B1 SMA (MRC Holland, Amsterdam, The Netherlands), according to the manufacturer’s protocol. MLPA copy-number classifications served as the reference for dPCR validation. MLPA data were analyzed using Coffalyser.Net (version v.260901.1020; MRC Holland, Amsterdam, The Netherlands).

*CFTR* p.Phe508del status was assessed by Sanger sequencing using the laboratory’s established diagnostic protocol. Sanger sequencing results served as the reference for evaluating dPCR concordance.

### 2.6. Population Screening Cohort

Between April and August 2026, 1670 women aged 20–35 years residing in Crete or Ikaria, Greece, participated in the screening program. Participation was voluntary, with no additional exclusion criteria, and all collected samples were analyzed. Following identification of female carriers, 10 male partners underwent targeted testing.

Laboratory staff organized informational meetings at local hospitals and health centers and trained healthcare professionals in pre-test counseling and DBS collection. Initial samples were collected during these visits, while subsequent sampling was performed locally, with DBS cards transported to Athens for analysis. Participants included women planning pregnancy and those in early pregnancy. Samples from pregnant women were prioritized to facilitate timely carrier notification, partner testing, and genetic counseling.

Pre-test information was provided through written leaflets and counseling by trained local staff. The leaflets described the screening targets, result notification procedures, availability of free partner testing for identified carriers, and an anticipated reporting time of ten days. Written informed consent was obtained through signatures on the DBS cards, as described in the Informed Consent Statement.

### 2.7. Post-Test Genetic Counseling and Return of Results

Screening results were provided through a secure online portal using the unique identification code assigned to each Guthrie card. Reports included an explanation of the findings, test limitations, and recommended follow-up.

Women identified as *SMN1* or *CFTR* p.Phe508del carriers were additionally notified by telephone and in writing. Genetic counseling was provided by a medical geneticist via telephone, videoconference, or in person at the Institute of Child Health, addressing reproductive implications, partner testing, and further genetic evaluation.

Counseling emphasized the residual carrier risk following a negative result. For SMA, a two-copy *SMN1* result reduced but did not exclude carrier status, with an estimated carrier detection rate of approximately 92%. For CF, a negative p.Phe508del result excluded only the targeted variant, with an estimated carrier detection rate of approximately 53% based on reported Greek *CFTR* allele frequencies. Expanded *CFTR* testing was recommended following negative screening results.

For partners of women carrying p.Phe508del, comprehensive *CFTR* analysis was recommended following a negative targeted test. Couples in which both partners were carriers of the same condition received counseling regarding prenatal diagnosis and in vitro fertilization (IVF) with preimplantation genetic testing for monogenic disorders (PGT-M).

### 2.8. Statistical Analysis

Continuous DNA concentrations and spectrophotometric purity ratios were summarized using descriptive statistics. The association between *RPPH1* copy number and spectrophotometric DNA concentration was assessed using Spearman′s rank correlation.

For *SMN1* and *SMN2*, dPCR measurements were compared with MLPA-defined copy-number classifications using receiver operating characteristic (ROC) analysis at each classification boundary. As adjacent copy-number groups did not overlap, ROC-derived cut-offs were defined as the midpoints between the highest measurement of the lower group and the lowest measurement of the higher group. Any threshold within these gaps maximized the observed Youden index (J = sensitivity + specificity − 1). Prespecified cut-offs of 0.5, 1.5, and 2.5, established before analysis of the validation dataset, were also evaluated.

Sensitivity, specificity, and overall agreement with the reference methods were calculated with exact (Clopper–Pearson) 95% confidence intervals. Categorical agreement between dPCR and MLPA was additionally assessed using Cohen′s κ. Because the ROC-derived cut-offs were determined and evaluated in the same validation dataset, their performance estimates are apparent and potentially optimistic. The prespecified cut-offs were evaluated in this same dataset and therefore also require independent validation.

Repeatability was evaluated using eight samples with known *SMN1* and *SMN2* copy-number genotypes, each independently eluted from DBS three times and analyzed by dPCR. For each sample and target, the mean, standard deviation (SD), and coefficient of variation (CV%) were calculated. Pooled CVs were calculated as the root mean square of the individual sample CVs. Exact 95% confidence intervals were also calculated for the proportions of concordant classifications.

### 2.9. Use of Generative AI

**Figure 1:**
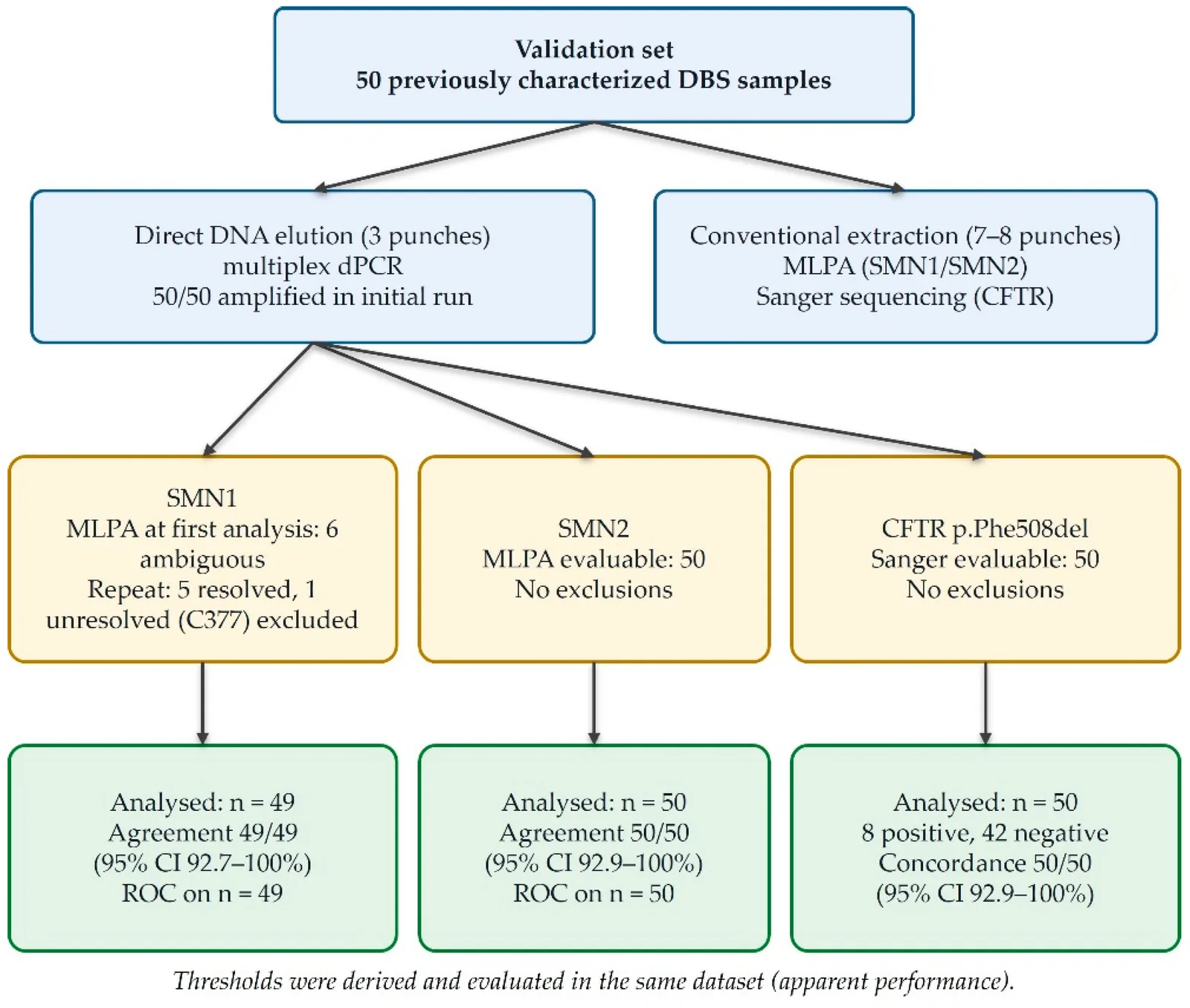
Flow of the 50 validation samples through direct-elution dPCR and reference-method analyses. Sample C377 yielded an indeterminate *SMN1* MLPA result and was excluded from the *SMN1* concordance and ROC analyses only.

**Figure 2:**
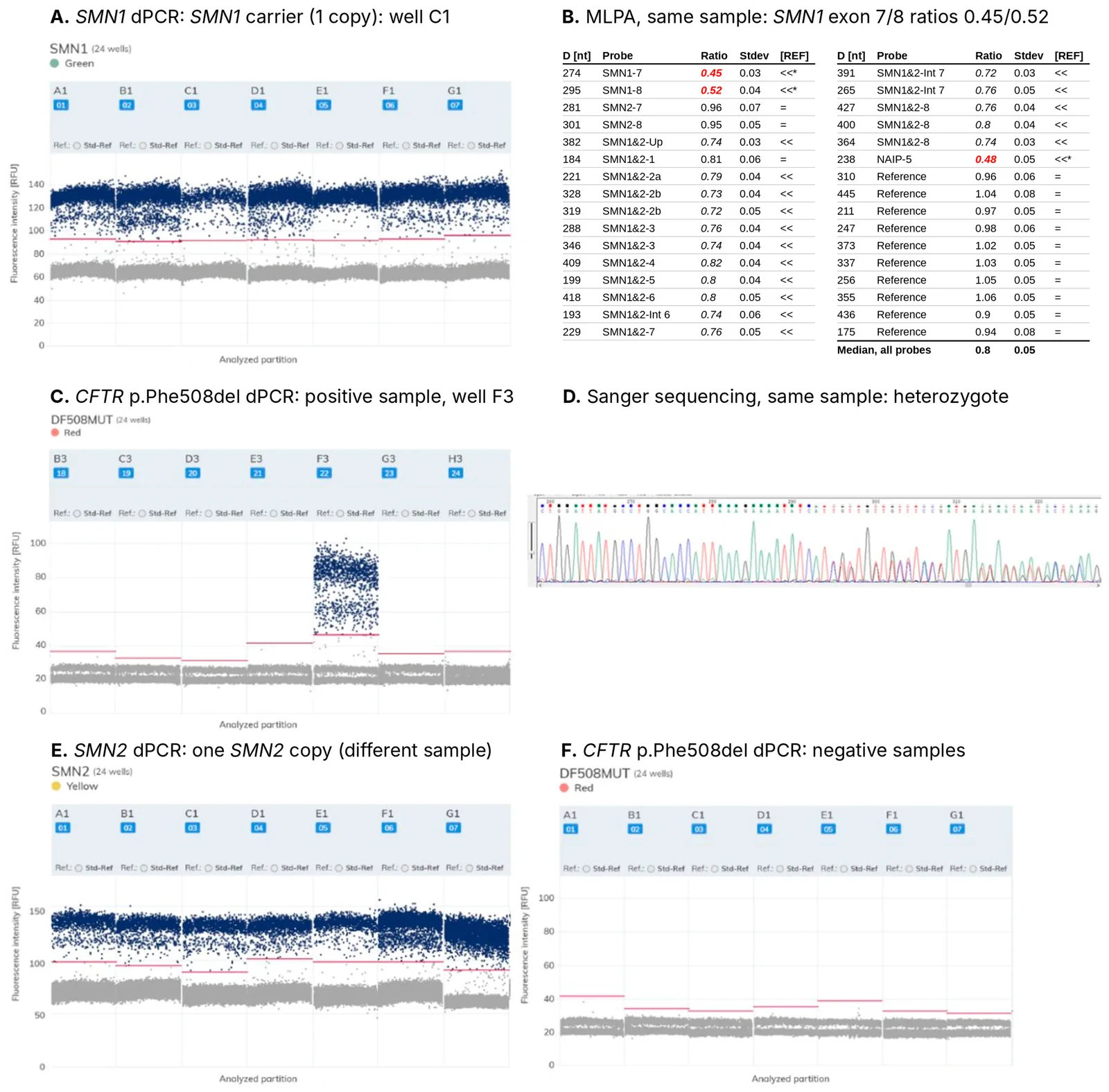
Representative dPCR and reference-method results. (**A**,**B**) *SMN1* dPCR amplitude plot (**A**) and corresponding MLPA report ((**B**); *SMN1* exon 7 and exon 8 ratios of 0.45 and 0.52, respectively; ratios outside the normal range, including the *NAIP* exon 5 probe, are highlighted in red/bold by Coffalyser.Net) from an *SMN1* carrier. Positive partitions are located above the fluorescence threshold (red line). (**C**,**D**) dPCR detection of *CFTR* p.Phe508del in a positive sample ((**C**), well F3) and corresponding Sanger sequencing showing heterozygosity (**D**). (**E**) *SMN2* dPCR showing one copy in a different sample. (**F**) *CFTR* p.Phe508del dPCR results from negative samples.

During manuscript preparation and revision, Claude (Claude Sonnet 5.5; Anthropic, San Francisco, CA, USA) and ChatGPT (GPT-6; OpenAI, San Francisco, CA, USA) were used to assist with English language editing, improving readability and text organization, calculation of summary statistics (exact confidence intervals, repeatability metrics and *RPPH1* copy numbers) from the authors’ own data, preparation of Supplementary Tables S13 and S14 and Figure 1, and assembly of Figure 2 from the authors’ original images.

These tools were not used to generate experimental data or develop the study methodology. All AI-assisted outputs were reviewed and verified by the authors, who take full responsibility for the scientific content of the manuscript.

## 3. Results

### 3.1. Validation Sample Flow

The flow of the 50 validation samples through direct-elution dPCR and reference-method analyses is summarized in Figure 1. All samples were successfully analyzed by dPCR in the initial run. Of the six samples with initially ambiguous *SMN1* MLPA results, five were resolved upon repeat testing, while one (C377) remained indeterminate, leaving 49 samples for *SMN1* concordance and ROC analyses. All 50 samples were included in the *SMN2* and *CFTR* p.Phe508del analyses.

### 3.2. DNA Yield and Purity of Direct Eluates and Extracted DNA (Validation Samples)

In the 50 validation samples, directly eluted DNA had a mean apparent concentration of 248.8 ± 99.2 ng/µL (range 17.4–538.7 ng/µL), with mean A260/A280 and A260/A230 ratios of 0.83 ± 0.08 and 0.61 ± 0.33, respectively. Conventionally extracted DNA had a mean concentration of 78.2 ± 27.5 ng/µL (range 35.9–130.6 ng/µL), with corresponding ratios of 1.47 ± 0.07 and 0.39 ± 0.04 (Supplementary Tables S1, S2 and S7).

Direct elution yielded higher apparent DNA concentrations but lower A260/A280 ratios than conventional extraction. However, spectrophotometric measurements of crude eluates may be influenced by non-nucleic-acid components and should not be interpreted as verified DNA yields. In the 50 directly eluted samples, the median *RPPH1* copy number was 5136 per 40 µL reaction (approximately 2570 diploid genome equivalents; range 2481–9944 copies). A weak positive correlation was observed between *RPPH1* copy number and spectrophotometric DNA concentration (Spearman′s ρ = 0.24; Supplementary Table S1).

### 3.3. Concordance with Reference Methods

For *SMN1*, sample C377 remained indeterminate by MLPA after repeat testing. Although dPCR indicated two *SMN1* copies (1.77), two independent MLPA analyses yielded intermediate exon 7 ratios (0.64 and 0.72), between the expected values for one and two copies, and an exon 8 ratio of 0.88 in both analyses. As no definitive MLPA copy-number classification could be assigned, C377 was excluded from the *SMN1* concordance analysis. Among the remaining 49 samples, dPCR and MLPA showed complete agreement (49/49; 100%; exact 95% CI 92.7–100%; Cohen′s κ = 1.00; Supplementary Table S8).

For *SMN2*, dPCR and MLPA classifications agreed in all 50 samples (50/50; 100%; exact 95% CI 92.9–100%; Cohen′s κ = 1.00; Supplementary Table S9). Individual *SMN1* and *SMN2* measurements are presented in Table 1.

For *CFTR* p.Phe508del, complete concordance was observed between dPCR and Sanger sequencing (50/50; exact 95% CI 92.9–100%). The observed sensitivity was 100% (8/8; 95% CI 63.1–100%) and specificity was 100% (42/42; 95% CI 91.6–100%). All eight positive samples were heterozygous; homozygous p.Phe508del samples were not included in the validation cohort (Supplementary Table S10). Representative dPCR, MLPA, and Sanger sequencing results are shown in Figure 2.

### 3.4. ROC Analysis and Classification Performance

ROC analysis demonstrated complete separation between adjacent *SMN1* and *SMN2* copy-number categories in the validation cohort. For each classification boundary (0 vs. ≥1, ≤1 vs. ≥2, and ≤2 vs. 3 copies), the area under the ROC curve (AUC) and Youden index were 1.000, with observed sensitivity and specificity of 100% (Table 2 and Table 3). Sample C377 was excluded from the *SMN1* analyses.

The ROC-derived cut-offs were 0.47, 1.39, and 2.56 for *SMN1*, and 0.42, 1.51, and 2.67 for *SMN2*, corresponding to the midpoints between adjacent non-overlapping copy-number groups. These cut-offs were not uniquely optimal, as any threshold within the corresponding gaps yielded identical classification performance. The gaps ranged from 0.53 to 0.97 (Supplementary Table S14), with no sample closer than 0.26 to a derived cut-off. Importantly, prespecified cut-offs of 0.5, 1.5, and 2.5, established before analysis of the validation dataset, produced identical classifications for all evaluable samples (49 for *SMN1* and 50 for *SMN2*).

Because the ROC-derived cut-offs were established and evaluated using the same validation dataset, their performance estimates are apparent and potentially optimistic. Although the prespecified cut-offs yielded identical results, their performance also requires independent validation. Confidence intervals were particularly wide for categories represented by only three samples (*SMN1* zero-copy and *SMN2* three-copy categories; exact 95% CI 29.2–100%; Supplementary Table S13), highlighting the limited precision of these estimates.

### 3.5. Repeatability of dPCR Measurements

Repeatability was evaluated using eight reference samples, each subjected to three independent DBS elutions followed by dPCR analysis. For *SMN1*, mean copy-number estimates ranged from 0.998 to 3.185, with coefficients of variation (CVs) of 1.3–4.8% and a pooled CV of 2.6% (Supplementary Table S11). For *SMN2*, mean estimates ranged from 0.879 to 2.074, with CVs of 0.34–4.23% and a pooled CV of 2.3% (Supplementary Table S12).

All eight samples maintained their expected copy-number classifications across the three independent preparations for both targets (8/8 samples per target; exact 95% CI 63–100%). All 24 measurements per target were correctly classified (24/24; exact 95% CI 86–100%), and mean copy-number estimates deviated from the expected integer values by no more than 0.22 copies.

### 3.6. Population Carrier Screening

Among 1670 women screened, 34 were identified as *SMN1* deletion carriers with one *SMN1* copy (2.04%; approximately 1 in 49; 95% CI 1.4–2.8%), and 20 as *CFTR* p.Phe508del carriers (1.20%; approximately 1 in 83; 95% CI 0.7–1.8%). One woman carried both findings, resulting in 53 individual female carriers. The *CFTR* carrier frequency refers exclusively to p.Phe508del. Eight of the 34 *SMN1* carriers and two of the 20 p.Phe508del carriers were pregnant.

All positive screening results were reproduced using new DNA preparations and confirmed by MLPA for *SMN1* (34/34; all with one copy; exact 95% CI 89.7–100%) or Sanger sequencing for *CFTR* p.Phe508del (20/20; exact 95% CI 83.2–100%). No unconfirmed positive results remained. No participants with zero *SMN1* copies or homozygous p.Phe508del were identified. Screen-negative results were not independently verified; therefore, the false-negative rate in the screening cohort could not be determined.

Repeat analysis following a new DNA elution was required for 55 samples (3.3%): 52 because of fewer than 18,000 valid partitions and three because of *RPPH1* amplification failure. All yielded valid results upon repeat testing.

All partners of identified carriers were informed about the availability of testing, but only 10 male partners submitted DBS cards. Two were confirmed as carriers, one for an *SMN1* deletion and one for *CFTR* p.Phe508del, identifying one SMA carrier couple and one CF carrier couple. Both couples received genetic counseling regarding reproductive options, including prenatal diagnosis and preimplantation genetic testing. The remaining eight partners tested negative for the investigated targets.

Operationally, up to two 24-well nanoplates could be processed daily, corresponding to a maximum of 48 reactions, including controls. Hands-on time for DBS punching, DNA elution, and plate preparation was approximately 1.5 h per plate. Screening results were generally reported within 10 days of sample receipt, extending to 15 days when confirmatory testing was required. The direct-elution multiplex workflow eliminated the need for conventional DNA extraction and capillary electrophoresis during initial screening; however, no formal cost comparison was performed.

## 4. Discussion

This study describes the development and preliminary validation of a dried blood spot (DBS)-based workflow combining direct DNA elution with multiplex digital PCR (dPCR) for *SMN1* and *SMN2* copy-number analysis and targeted detection of *CFTR* p.Phe508del. The workflow was subsequently applied to a screening cohort of 1670 women, followed by targeted testing of reproductive partners of identified carriers.

Direct DNA elution from DBS simplified sample preparation by eliminating conventional DNA extraction before dPCR analysis. All 50 validation samples yielded amplifiable DNA, supporting the technical feasibility of this approach across the investigated targets. However, conventional extraction was used to prepare DNA for the reference methods, and the two workflows were not formally compared for accuracy or efficiency; therefore, superiority of direct elution cannot be inferred. Nevertheless, simplified sample preparation, together with the logistical advantages of DBS collection, storage, transport, and centralized processing, may facilitate population-based molecular screening [18].

Spectrophotometric measurements of directly eluted DNA require cautious interpretation. The mean A260/A280 ratio (0.83) was substantially below that expected for purified DNA, while A260/A230 ratios were low in both preparations. These findings suggest interference from non-nucleic-acid components, potentially including proteins, hemoglobin-derived material, and buffer constituents. Consequently, the higher apparent DNA concentrations measured after direct elution should not be interpreted as greater DNA recovery, particularly because DNA concentrations were not independently verified using a DNA-specific method. The suitability of the eluates for dPCR was instead supported by successful target amplification, including the *RPPH1* reference target, although inhibition was not specifically evaluated.

The multiplex assay demonstrated complete concordance with MLPA for *SMN1* and *SMN2* copy-number classifications and with Sanger sequencing for *CFTR* p.Phe508del detection in the evaluable validation samples. *SMN1* exon 7 dosage analysis is an established component of SMA testing, while *SMN2* copy number provides information relevant to disease severity [19,20]. The proposed assay is intended for first-tier reproductive carrier screening rather than diagnosis of affected individuals, and positive screening findings require confirmation using an independent method. Repeatability across three independent DBS preparations showed coefficients of variation below 5% for both *SMN1* and *SMN2*, although these estimates were based on only eight samples. Prespecified cut-offs of 0.5, 1.5, and 2.5 produced classifications identical to those obtained using ROC-derived thresholds, reducing concerns about dependence on fitted values. Nevertheless, independent validation remains necessary. This is consistent with the emphasis of the digital MIQE guidelines on transparent reporting and reproducibility of dPCR experiments [21].

The observed *SMN1* carrier frequency was 2.04% (1 in 49; 95% CI 1.4–2.8%), comparable to the previously reported Greek frequency of 2.3% (1 in 43) [5]. The earlier Greek study also highlighted the potential contribution of systematic carrier screening to SMA prevention, drawing parallels with established hemoglobinopathy prevention programs [5]. Similarly, a Chinese study involving 39,647 women reported an *SMN1* carrier frequency of 1.83% [22]. Differences in participant characteristics and screening methods should be considered when comparing these estimates.

An important limitation of *SMN1* copy-number screening is the residual carrier risk following a two-copy result. The assay cannot identify silent carriers with two *SMN1* copies on one chromosome and none on the other (2 + 0), or carriers of certain pathogenic *SMN1* sequence variants. Reported carrier detection rates vary across populations, including 91.2% in a pan-ethnic cohort, 94.8% in individuals of European ancestry, and 70.5% in African Americans [23]. An Australian estimate places the residual carrier risk after a two-copy result in individuals without a family history of SMA at approximately 1 in 650 [2], although this estimate may not apply to other populations. Analysis of *SMN1* duplication-associated markers, including c.*3+80T>G and c.211_212del, can further refine residual-risk assessment [24]. Moreover, a study of parents of affected children found that 9 of 118 (7.6%) carried two *SMN1* copies and would not have been identified through conventional copy-number screening alone [25]. Thus, a two-copy result reduces but does not eliminate carrier risk; the population-specific residual risk in Greece remains to be established.

The *CFTR* component is limited to targeted p.Phe508del detection and does not constitute comprehensive CF carrier screening. This variant accounts for approximately 53% of CF alleles in Greece [8,9], leaving a substantial proportion of carriers undetected. In a Greek exome-sequencing cohort, p.Phe508del was identified in 1.9% of individuals, whereas pathogenic *CFTR* variants overall were detected in 4.2% [7]. Based on these findings and the observed p.Phe508del carrier frequency in the present study, illustrative estimates of residual carrier risk after a negative targeted result range from approximately 1.1% to 2.3% (about 1 in 90 to 1 in 43). These estimates depend on assumptions regarding variant distribution and population representativeness and should not be interpreted as validated population-specific risks. The exome cohort predominantly included clinically referred children and may not represent the general Greek population. More comprehensive *CFTR* analysis, including sequencing and deletion/duplication testing, has been reported to detect approximately 95% of carriers in Greece [4], while current ACMG recommendations support broader variant coverage than single-variant screening [10]. Comprehensive *CFTR* analysis should therefore be offered to partners of identified carriers and considered when family history or other clinical indications warrant further investigation.

The screening workflow incorporated result reporting, genetic counseling, and targeted partner testing. Among the 53 women identified as carriers of at least one investigated condition, 10 partners underwent testing, leading to the identification of one SMA carrier couple and one CF carrier couple. These findings illustrate the potential reproductive relevance of the screening pathway, although partner-testing uptake was limited.

Within the ACCE framework for evaluating genetic tests [26], this study primarily addresses analytical validity through comparison with established reference methods and demonstrates the technical feasibility of an integrated screening and counseling pathway. The observed carrier frequencies and identification of carrier couples provide preliminary information relevant to screening implementation but do not establish clinical validity or clinical utility. Clinical outcomes, reproductive decisions, psychosocial effects, long-term follow-up, and cost-effectiveness were not evaluated. Consequently, the identification of carrier couples should not be interpreted as evidence of improved reproductive outcomes.

Several limitations should be acknowledged. Analytical validation involved only 50 previously characterized samples, with limited representation of some categories, including three *SMN1* zero-copy samples and eight heterozygous p.Phe508del-positive samples. Homozygous p.Phe508del and certain rare copy-number configurations were not evaluated. Although concordance was complete, confidence intervals were wide, particularly for smaller categories. One sample (C377) yielded persistently intermediate *SMN1* MLPA ratios and could not be assigned a definitive reference copy number, illustrating the challenges of interpreting indeterminate reference results. In addition, ROC-derived thresholds were established and evaluated in the same dataset and were not independently validated, repeatability was assessed in only eight samples, and comparisons with MLPA and Sanger sequencing demonstrate agreement with those methods rather than absolute analytical equivalence. Broader validation across laboratories, operators, instruments, and sample conditions is therefore required. The applicability of direct DBS elution to more extensive *CFTR* analysis, sequencing, and assays requiring longer DNA fragments also remains to be evaluated.

Finally, the screening cohort was restricted to women aged 20–35 years from two Greek islands, and all analyses were performed in a single laboratory using one dPCR platform, limiting generalizability. Operationally, up to two 24-well nanoplates could be processed daily, corresponding to a maximum of 48 reactions, including controls, with approximately 1.5 h of hands-on time per plate. Results were generally reported within 10 days of sample receipt, extending to 15 days when confirmatory testing was required. Although direct elution reduced sample-processing steps, no formal comparison of throughput, turnaround time, or cost with extraction-based or sequencing-based workflows was performed. Future evaluations should also consider the broader costs of genetic counseling, partner testing, and clinical follow-up [13].

## 5. Conclusions

This study developed and preliminarily validated a dried blood spot (DBS)-based workflow combining direct DNA elution with multiplex digital PCR (dPCR) for *SMN1* and *SMN2* copy-number analysis and targeted detection of *CFTR* p.Phe508del. The workflow showed complete concordance with reference methods in a limited validation cohort and was successfully implemented in a screening cohort of 1670 women. Partner testing identified two carrier couples, demonstrating the practical integration of molecular screening, confirmatory testing, and genetic counseling.

These findings support the further development of a structured, publicly supported reproductive carrier screening program in Greece, building on existing screening infrastructure. However, the targeted nature of the assay leaves residual carrier risk, which must be addressed through appropriate counseling. Larger independent validation, broader *CFTR* variant coverage, and systematic evaluation of screening uptake, operational performance, cost-effectiveness, and clinical outcomes are needed to guide implementation within routine public health services.

## Figures and Tables

**Table 1:** Comparison of *SMN1/SMN2* copy number determination with dPCR and MLPA.

Case N^o^	dPCR		dPCR (Copy N^o^)		MLPA	MLPA	MLPA	MLPA	MLPA (Copy N^o^)			
	*SMN1* EX7-8	*SMN2* EX7-8	*SMN1*	*SMN2*	*SMN1* EX7	*SMN1* EX8	*SMN2* EX7	*SMN2* EX8	*SMN1*		*SMN2*	
									EX7	EX8	EX7	EX8
C672	3.2310	1.2469	3	1	1.52	1.64	0.41	0.42	3	3	1	1
C61	3.1246	1.0697	3	1	1.57	1.63	0.41	0.45	3	3	1	1
C91	3.0463	0.0029	3	0	1.64	1.64	0	0	3	3	0	0
C86	1.7894	0.9049	2	1	1.04	1.19	0.41	0.41	2	2	1	1
C95	3.1290	1.0240	3	1	1.62	1.64	0.42	0.43	3	3	1	1
C736	0.9883	1.9639	1	2	0.58	0.58	0.85	0.91	1	1	2	2
C493	2.1201	1.041	2	1	1.18	1.17	0.5	0.51	2	2	1	1
C721	2.0449	0.0034	2	0	1.19	1.13	0	0	2	2	0	0
C505	1.7741	0.8805	2	1	0.97	0.8	0.48	0.51	2	2	1	1
C751	1.0683	3.1617	1	3	0.41	0.41	1	1.39	1	1	3	3
C515	1.7950	1.8640	2	2	1.07	0.87	0.99	0.99	2	2	2	2
C518	1.7471	1.7719	2	2	1.02	0.89	0.91	0.98	2	2	2	2
C527	1.7078	0.9242	2	1	0.93	0.96	0.48	0.5	2	2	1	1
C693	1.7763	1.8808	2	2	0.91	0.98	0.87	0.97	2	2	2	2
C563	1.6847	0.8594	2	1	1	0.98	0.52	0.55	2	2	1	1
C1138	2.0145	2.0668	2	2	1.32	1.36	1.18	1.08	2	2	2	2
C1143	2.0040	2.0282	2	2	1.12	1.02	1.14	1	2	2	2	2
C1141	0	1.9602	0	2	0	0	1.12	1.04	0	0	2	2
C1114	2.0294	1.9757	2	2	1.15	1.03	1.19	1.06	2	2	2	2
C461	0.9655	1.9355	1	2	0.63	0.58	1.05	1.03	1	1	2	2
C942	0.9300	0.9489	1	1	0.47	0.52	0.45	0.52	1	1	1	1
C1067	0.9306	0	1	0	0.53	0.51	0	0	1	1	0	0
C860	1.0629	2.1741	1	2	0.45	0.52	0.96	0.95	1	1	2	2
C870	1.0666	1.9541	1	2	0.64	0.64	1.16	0.98	1	1	2	2
C892	1.8582	1.8097	2	2	0.83	0.92	0.83	0.91	2	2	2	2
C571	1.8445	1.8917	2	2	0.87	0.94	0.85	1	2	2	2	2
C955	1.7627	0.9152	2	1	0.92	0.87	0.42	0.53	2	2	1	1
C373	2.9944	0.0001	3	0	1.46	1.39	0	0	3	3	0	0
C377	1.7673	0.9815	2	1	0.72	0.88	0.41	0.45	ambiguous	2	1	1
C36	1.0616	2.0802	1	2	0.63	0.5	1.09	1.08	1	1	2	2
C150	1.0429	0.9815	1	1	0.56	0.58	0.47	0.51	1	1	1	1
C258	1.9444	0.0011	2	0	1.18	1.14	0	0	2	2	0	0
C601	0.0015	1.8598	0	2	0	0	0.89	0.92	0	0	2	2
C547	1.9632	1.0567	2	1	0.98	1.02	0.45	0.48	2	2	1	1
C1000	0.9868	2.0905	1	2	0.52	0.59	1.11	1.15	1	1	2	2
C852	0.0009	3.3748	0	3	0	0	1.52	1.64	0	0	3	3
C457	1.9037	0.8997	2	1	0.97	1.18	0.42	0.47	2	2	1	1
C321	1.0893	2.1880	1	2	0.49	0.52	1.17	1.09	1	1	2	2
C587	1.9151	0.8364	2	1	0.96	1.08	0.43	0.47	2	2	1	1
C459	1.9160	1.9119	2	2	1.04	0.99	0.85	1.02	2	2	2	2
C159	0.9320	0.9481	1	1	0.55	0.48	0.58	0.62	1	1	1	1
C200	1.9537	0.9571	2	1	0.93	1.06	0.61	0.64	2	2	1	1
C351	1.9201	1.9721	2	2	0.99	1.02	1.07	0.91	2	2	2	2
C657	1.9499	1.9650	2	2	1.11	1.05	0.85	0.91	2	2	2	2
C254	2.0348	2.0361	2	2	1.07	1.12	0.97	1.02	2	2	2	2
C872	2.0457	1.9942	2	2	1.06	0.91	0.89	0.97	2	2	2	2
C367	1.0681	3.1628	1	3	0.48	0.53	1.35	1.48	1	1	3	3
C453	1.8902	0.962	2	1	0.85	0.92	0.43	0.49	2	2	1	1
C479	2.0110	1.9126	2	2	1.12	1.09	0.94	1.03	2	2	2	2
C234	1.0419	0.9729	1	1	0.51	0.59	0.48	0.61	1	1	1	1

Abbreviations: dPCR, Digital Polymerase Chain Reaction; MLPA, Multiplex Ligation-Dependent Probe Amplification; *SMN1*, Survival Motor Neuron 1; *SMN2*, Survival Motor Neuron 2; EX, exon.

**Table 2:** *SMN1*—dPCR thresholds and diagnostic performance verified against MLPA.

Comparison	AUC	Cut-Off *	Sensitivity	Specificity	Youden J
0 vs. ≥1 copies	1.000	0.47	100%	100%	1.000
≤1 vs. ≥2 copies	1.000	1.39	100%	100%	1.000
≤2 vs. 3 copies	1.000	2.56	100%	100%	1.000

* Cut-offs are the midpoints of the gap between adjacent copy-number groups (any value in the gap gives J = 1.000) in the validation cohort and were evaluated in the same cohort (apparent performance); exact 95% confidence intervals are given in Supplementary Table S13. One sample (C377) with an indeterminate MLPA result was excluded from the *SMN1* concordance and ROC analyses. Abbreviations: AUC, area under the ROC curve; dPCR, digital PCR; MLPA, multiplex ligation-dependent probe amplification; ROC, receiver operating characteristic; J, Youden index.

**Table 3:** *SMN2*—dPCR thresholds and diagnostic performance verified against MLPA.

Comparison	AUC	Cut-Off *	Sensitivity	Specificity	Youden J
0 vs. ≥1 copies	1.000	0.42	100%	100%	1.000
≤1 vs. ≥2 copies	1.000	1.51	100%	100%	1.000
≤2 vs. 3 copies	1.000	2.67	100%	100%	1.000

* Cut-offs are the midpoints of the gap between adjacent copy-number groups (any value in the gap gives J = 1.000) in the validation cohort and were evaluated in the same cohort (apparent performance); exact 95% confidence intervals are given in Supplementary Table S13. Abbreviations: AUC, area under the ROC curve; dPCR, digital PCR; MLPA, multiplex ligation-dependent probe amplification; ROC, receiver operating characteristic; J, Youden index.

## Data Availability

The data presented in this study are not publicly available due to privacy and ethical restrictions concerning the protection of participants’ personal and genetic data. The data may be available from the corresponding author upon reasonable request and subject to approval by the relevant institutional authorities and applicable data protection regulations.

## Supplementary Materials

The following supporting information can be downloaded at https://www.mdpi.com/article/10.3390/diagnostics16213538/s1, Table S1: DNA concentrations measured in the 50 samples following elution for digital PCR analysis; *RPPH1* values are from the dPCR reaction (40 µL, containing 2 µL of eluate); Table S2: DNA concentrations measured in the 50 samples following extraction with commercially available kits; Table S3: Sequences of primers and hydrolysis probes used for digital PCR assays; Table S4: Preparation of 10× primer–probe assay mixes used in the multiplex digital PCR reaction; Table S5: Multiplex Digital PCR reaction mixture; Table S6: Imaging parameters used for digital PCR fluorescence acquisition; Table S7: Summary statistics of DNA concentration and purity measurements in samples analyzed by dPCR and MLPA; Table S8: Concordance between dPCR and MLPA for *SMN1* copy-number determination; Table S9: Concordance between dPCR and MLPA for *SMN2* copy-number determination; Table S10: Results from dPCR in comparison with the Sanger sequencing for *CFTR* p.Phe508del variant; Table S11: Repeatability of *SMN1* dPCR measurements across three independent DNA preparations (separate elutions); Table S12: Repeatability of *SMN2* dPCR measurements across three independent DNA preparations (separate elutions); Table S13: Exact (Clopper–Pearson) 95% confidence intervals for the proportion of samples correctly classified by dPCR in the validation set; Table S14: Gaps between adjacent copy-number groups and resulting dPCR cut-offs (validation set).
