# Supplementary material for: DBS Direct Elution and Multiplex Digital PCR for SMN1 and SMN2 Copy-Number and CFTR Variant Detection: Development and Validation of a Molecular Screening Workflow
Source: Diagnostics (Basel). 2026 Oct 28;16(21):3538. doi: 10.3390/diagnostics16213538 (PMC13761612; doi:10.3390/diagnostics16213538)
Supplement: Supplementary file 1 [file diagnostics-16-03538-s001.zip › diagnostics-4631285-supplementary.pdf]

Supplementary Table S1

DNA concentrations measured in the 50 samples following elution for digital PCR analysis; RPPH1 values are from the dPCR reaction (40 μL, containing 2 μL of eluate)

dPCR elution	Concentration (ng/μL)	A260/A280	A260/A230	RPPH1 (copies/μL, reaction)	RPPH1 (copies/reaction)
C672	45.6	1.17	0.6	98.5	3938
C61	200	0.96	2.3	79.6	3182
C91	17.4	1.26	0.33	108.1	4324
C86	164.8	0.82	0.53	74.4	2976
C95	201	0.84	0.7	72.6	2906
C736	390	0.81	1.19	205.6	8224
C493	178.5	0.81	0.54	128.0	5120
C721	160	0.82	0.51	174.1	6964
C505	108	0.82	0.54	199.6	7984
C751	240	0.8	0.58	122.4	4896
C515	183.5	0.77	0.53	178.3	7132
C518	331.3	0.82	0.84	171.5	6860
C527	266.5	0.79	0.76	102.1	4084
C693	171	0.82	0.4	151.2	6048
C563	192.8	0.79	0.59	84.6	3384
C1138	184.4	0.82	0.58	123.1	4924
C1143	195.8	0.79	0.57	167.7	6708
C1141	177.8	0.79	0.54	84.1	3362
C1114	327.9	0.8	0.94	128.8	5152
C461	166.1	0.83	0.41	75.6	3024
C942	213.6	0.82	0.44	145.8	5832
C1067	315.6	0.82	0.63	108.0	4320
C860	212.4	0.79	0.43	134.9	5396
C870	302.4	0.83	0.19	74.9	2995
C892	389.1	0.81	1.05	72.5	2902
C571	262.5	0.79	0.5	132.8	5312
C955	221.5	0.83	0.44	217.5	8700
C373	538.7	0.79	0.21	239.2	9568
C377	201.9	0.8	0.41	138.9	5556
C36	317	0.81	0.66	171.7	6868
C150	456.3	0.81	1.26	150.1	6004
C258	319.2	0.82	0.67	185.6	7424
C601	353.9	0.81	0.73	183.9	7356
C547	284.8	0.81	0.59	207.4	8296
C1000	307.6	0.83	0.6	159.2	6368
C852	223.6	0.82	0.4	144.3	5772
C457	353.6	0.79	0.77	138.6	5544
C321	345.5	0.82	0.73	62.0	2481
C587	370.8	0.82	0.85	113.3	4532
C459	147.3	0.82	0.36	108.3	4332
C159	365.1	0.82	0.2	248.6	9944
C200	238.5	0.81	0.48	113.8	4552
C351	171	0.82	0.4	86.4	3457
C657	158	0.82	0.39	124.1	4964
C254	196.2	0.82	0.42	108.4	4336
C872	216.2	0.81	0.44	88.3	3532
C367	311.5	0.81	0.67	188.0	7520
C453	321.8	0.8	0.73	83.8	3352
C479	238.3	0.81	0.49	90.2	3608
C234	181.3	0.85	0.41	149.4	5976

Supplementary Table S2

DNA concentrations measured in the 50 samples following extraction with commercially available kits

7-8 Punches	Concentration (ng/µL)	A260/280	A260/230
C672	48.1	1.49	0.32
C61	126.8	1.43	0.39
C91	45.9	1.47	0.3
C86	55.6	1.46	0.36
C95	48.7	1.41	0.34
C736	67.5	1.52	0.39
C493	85.1	1.42	0.39
C721	70.9	1.45	0.38
C505	90	1.4	0.37
C751	125.8	1.33	0.38
C515	63.3	1.48	0.35
C518	54.3	1.53	0.36
C527	78.1	1.44	0.39
C693	63.2	1.4	0.35
C563	62.8	1.5	0.36
C1138	91	1.32	0.35
C1143	48	1.4	0.31
C1141	74.1	1.46	0.39
C1114	88.9	1.56	0.44
C461	115.5	1.5	0.42
C942	79.1	1.41	0.36
C1067	79.9	1.46	0.35
C860	93.7	1.46	0.41
C870	94.1	1.52	0.46
C892	128	1.46	0.43
C571	126.7	1.33	0.37
C955	106.6	1.43	0.4
C373	77	1.47	0.36
C377	106.6	1.43	0.42
C36	79.1	1.47	0.47
C150	48.9	1.58	0.39
C258	38.1	1.55	0.42
C601	48.2	1.56	0.44
C547	35.9	1.47	0.34
C1000	55.8	1.49	0.39
C852	43.5	1.56	0.44
C457	49	1.51	0.38
C321	54	1.5	0.4
C587	42	1.55	0.39
C459	54.2	1.41	0.39
C159	64	1.54	0.45
C200	70.2	1.47	0.48
C351	106.5	1.51	0.35
C657	130.6	1.56	0.47
C254	95.5	1.55	0.36
C872	125.3	1.58	0.35
C367	85.4	1.33	0.39
C453	80.1	1.4	0.44
C479	98.7	1.5	0.46
C234	107.2	1.57	0.4

Supplementary Table S3
Sequences of primers and hydrolysis probes used for digital PCR assays

Target	Primer/Probe	Sequence (5'→3')	Fluorophore
CFTR p.Phe508del	Forward	TTGATAATGACCTAATAATGATGGGTT	
	Reverse	GTGAAGGGTTCATATGCATAATCAA	
	Probe	AAT CAT CGG TGT TTC CTC A	ROX
SMN1/*SMN2*	Forward	AAT GCT TTT TAA CAT CCA TAT AAA GCT	
	Reverse	CCT TAA TTT AAG GAA TGT GAG CAC C	
SMN1	Probe	TCCTTACAGGGTTTCAGACAAAATCAA	FAM
SMN2	Probe	TCCTTACAGGGTTTTAGACAAAATCAA	HEX
RPPH1	Forward	CTT TGC CGG AGC TTG GA	
	Reverse	GAGAGTAGTCTGAATTGGGTTATGA	
	Probe	ACCTCACCTCAGCCATTGAACTCAC	Cy5

Probe sequences are shown in the 5′→3′ orientation. The corresponding reporter fluorophores are indicated in the final column. Probe modifications/quencher chemistry are retained according to the oligonucleotide supplier's nomenclature.

Supplementary Table S4
Preparation of 10× primer–probe assay mixes used in the multiplex digital PCR reaction

Assay	Component	Volume (µL)	Final concentration in 10× mix
***CFTR* p.Phe508del**	Nuclease-free water	176	–
	Primer pool	16	8 µM each primer
	CFTR p.Phe508del probe	8	4 µM
	**Total**	**200**	–
***SMN1*/*SMN2* multiplex**	Nuclease-free water	168	–
	Primer pool	16	8 µM each primer
	SMN1 probe	8	4 µM
	SMN2 probe	8	4 µM
	**Total**	**200**	–
RPPH1	Nuclease-free water	176	–
	Primer pool	16	8 µM each primer
	RPPH1 probe	8	4 µM
	**Total**	**200**	–

Supplementary Table S5
Multiplex Digital PCR reaction mixture

Component	Final volume (µL)
QIAcuity Probe Master Mix (4×)	10
Restriction Enzyme / EcoRI (20,000 units/ml)	1
SMN1/*SMN2* assay (10x)	4
CFTR p.Phe508del assay(10x)	4
RPPH1 assay(10x)	4
Water	15
DNA	2
**Total**	**40**

Supplementary Table S6

Imaging parameters used for digital PCR fluorescence acquisition

Channel	Exposure duration (ms)	Gain	Fluorophore
GREEN	300	6	FAM
YELLOW	300	6	HEX
RED	300	4	ROX
CRIMSON	150	4	Cy5

Supplementary Table S7

Summary statistics of DNA concentration and purity measurements in samples analyzed by dPCR and MLPA

Parameter	dPCR (n=50)	MLPA (n=50)
DNA concentration (ng/μL), mean ± SD	248.8 ± 99.2	78.2 ± 27.5
DNA concentration (ng/μL), median	222.6	77.6
DNA concentration (ng/μL), range	17.4–538.7	35.9–130.6
A260/A280, mean ± SD	0.83 ± 0.08	1.47 ± 0.07
A260/A280, median	0.82	1.47
A260/A280, range	0.77–1.26	1.32–1.58
A260/A230, mean ± SD	0.61 ± 0.33	0.39 ± 0.04
A260/A230, median	0.54	0.39
A260/A230, range	0.19–2.30	0.30–0.48

Supplementary Table S8

Concordance between dPCR and MLPA for *SMN1* copy-number determination

SMN1 copy number	dPCR, n	MLPA, n	Concordance
0	3	3	100%
1	14	14	100%
2	27	27	100%
3	5	5	100%
**Total**	49	49	100%
**Overall agreement:** 100% (49/49); **Cohen's κ = 1.00**.			

Supplementary Table S9

Concordance between dPCR and MLPA for *SMN2* copy-number determination

SMN2 copy number	dPCR, n	MLPA, n	Concordance
0	5	5	100%
1	19	19	100%
2	23	23	100%
3	3	3	100%
**Total**	50	50	100%

**Overall agreement:** 100% (50/50); **Cohen's κ = 1.00**

Supplementary Table S10

Results from dPCR in comparison with the Sanger sequencing for *CFTR* p.Phe508del variant

Case Nº	dPCR estimated copy number	Sanger Sequencing Result
C672	n.a.	wt/wt
C61	n.a.	wt/wt
C91	n.a.	wt/wt
C86	n.a.	wt/wt
C95	n.a.	wt/wt
C736	n.a.	wt/wt
C493	n.a.	wt/wt
C721	1.0512	**wt/mut**
C505	n.a.	wt/wt
C751	n.a.	wt/wt
C515	n.a.	wt/wt
C518	n.a.	wt/wt
C527	n.a.	wt/wt
C693	n.a.	wt/wt
C563	n.a.	wt/wt
C1138	n.a.	wt/wt
C1143	n.a.	wt/wt
C1141	n.a.	wt/wt
C1114	n.a.	wt/wt
C461	n.a.	wt/wt
C942	n.a.	wt/wt
C1067	n.a.	wt/wt
C860	n.a.	wt/wt
C870	n.a.	wt/wt
C892	0.9897	**wt/mut**
C571	n.a.	wt/wt
C955	1.1358	**wt/mut**
C373	n.a.	wt/wt
C377	n.a.	wt/wt
C36	n.a.	wt/wt
C150	n.a.	wt/wt
C258	n.a.	wt/wt
C601	n.a.	wt/wt
C547	0.9745	**wt/mut**
C1000	n.a.	wt/wt
C852	n.a.	wt/wt
C457	1.0504	**wt/mut**
C321	n.a.	wt/wt
C587	1.0984	**wt/mut**
C459	n.a.	wt/wt
C159	n.a.	wt/wt
C200	n.a.	wt/wt
C351	n.a.	wt/wt
C657	0.9735	**wt/mut**
C254	n.a.	wt/wt
C872	n.a.	wt/wt
C367	n.a.	wt/wt
C453	n.a.	wt/wt
C479	1.0557	**wt/mut**
C234	n.a.	wt/wt

n.a.: no quantifiable target signal detected

Supplementary Table S11.

Repeatability of *SMN1* dPCR measurements across three independent DNA preparations (separate elutions)

Sample	Reference *SMN*1 copies	Mean	SD	CV (%)
C672	3	3.185	0.054	1.7%
C150	1	1.027	0.026	2.6%
C515	2	1.861	0.089	4.8%
C200	2	2.015	0.060	3.0%
C872	2	1.995	0.026	1.3%
C587	2	1.968	0.046	2.4%
C351	2	1.988	0.031	1.6%
C1000	1	0.998	0.015	1.5%

Supplementary Table S12

Repeatability of *SMN2* dPCR measurements across three independent DNA preparations (separate elutions)

Sample	Reference *SMN2* copies	Mean	SD	CV (%)
C672	1	1.216	0.027	2.2%
C150	1	0.983	0.007	0.8%
C515	2	1.891	0.024	1.3%
C200	1	0.985	0.024	2.5%
C872	2	1.994	0.007	0.3%
C587	1	0.879	0.037	4.2%
C351	2	1.964	0.040	2.0%
C1000	2	2.074	0.056	2.7%

Supplementary Table S13

Exact (Clopper–Pearson) 95% confidence intervals for the proportion of samples correctly classified by dPCR in the validation set

Target	Reference class	Correctly classified	Proportion (%)	Exact 95% CI (%)
SMN1	0 copies (MLPA)	3/3	100	29.2–100.0
SMN1	1 copy (MLPA)	14/14	100	76.8–100.0
SMN1	2 copies (MLPA)	27/27	100	87.2–100.0
SMN1	3 copies (MLPA)	5/5	100	47.8–100.0
SMN1	All evaluable samples	49/49	100	92.7–100.0
SMN2	0 copies (MLPA)	5/5	100	47.8–100.0
SMN2	1 copy (MLPA)	19/19	100	82.4–100.0
SMN2	2 copies (MLPA)	23/23	100	85.2–100.0
SMN2	3 copies (MLPA)	3/3	100	29.2–100.0
SMN2	All evaluable samples	50/50	100	92.9–100.0
CFTR p.Phe508del	Positive (Sanger), sensitivity	8/8	100	63.1–100.0
CFTR p.Phe508del	Negative (Sanger), specificity	42/42	100	91.6–100.0

CFTR p.Phe508del	All samples	50/50	100	92.9–100.0
SMN1 repeatability	Samples with identical calls in 3 preparations	8/8	100	63.1–100.0
SMN1 repeatability	Measurements matching expected copy number	24/24	100	85.8–100.0
SMN2 repeatability	Samples with identical calls in 3 preparations	8/8	100	63.1–100.0
SMN2 repeatability	Measurements matching expected copy number	24/24	100	85.8–100.0

Thresholds were derived and evaluated in the same 50-sample dataset; values are apparent performance. SMN1 analysis excludes sample C377 (indeterminate MLPA result: SMN1 exon 7 ratios 0.64 and 0.72 in two independent analyses, between one and two copies, exon 8 ratio 0.88; dPCR two copies). The cut-offs are the midpoints of the gaps between adjacent groups (Table S14); cut-offs of 0.5, 1.5 and 2.5 give identical classification.

Supplementary Table S14

Gaps between adjacent copy-number groups and resulting dPCR cut-offs (validation set)

Marker, boundary	Highest value, lower group	Lowest value, upper group	Gap	Cut-off (midpoint)
SMN1, 0 vs 1 copy	0.0015	0.930	0.929	0.466
SMN1, 1 vs 2 copies	1.089	1.685	0.595	1.387
SMN1, 2 vs 3 copies	2.120	2.994	0.874	2.557
SMN2, 0 vs 1 copy	0.003	0.836	0.833	0.420
SMN2, 1 vs 2 copies	1.247	1.772	0.525	1.509
SMN2, 2 vs 3 copies	2.188	3.162	0.974	2.675
